# Feasibility of establishing a core set of sexual, reproductive, maternal, newborn, child, and adolescent health indicators in humanitarian settings: results from a multi-methods assessment in the Democratic Republic of Congo

**DOI:** 10.1186/s12978-022-01415-9

**Published:** 2022-06-02

**Authors:** Jacques Emina, Rinelle Etinkum, Anya Aissaoui, Cady Nyombe Gbomosa, Kaeshan Elamurugan, Kanya Lakshmi Rajendra, Ieman Mona El Mowafi, Loulou Kobeissi

**Affiliations:** 1 University of Kinshasa, Kinshasa, Democratic Republic of Congo; 2 Population and Health Research Institute, Kinshasa, Democratic Republic of Congo; 3 Faculty of Health Sciences, University of Ottawa, Ottawa, ON Canada; 4 NORImpact Consultancy AS, Rytterfaret 17A, Hafrsjord, Norway; 5 Institute for Population Health, University of Ottawa, Ottawa, ON Canada; 6 Cambridge Reproductive Health Consultants, Cambridge, MA USA; 7 Department of Sexual and Reproductive Health and Research (SRH), World Health Organization, Geneva, Switzerland

**Keywords:** The Democratic Republic of Congo, Monitoring and evaluating, Sexual and reproductive health, Maternal, child and adolescent health, Humanitarian data reporting, Health information systems, Refugees, Refugee health, République démocratique du Congo, Suivi et évaluation, Santé sexuelle et reproductive, Santé maternelle, infantile et adolescente, Communication de données humanitaires, Systèmes d'information sanitaire, Réfugiés, Santé des réfugiés, Den demokratiske republikken Kongo, Overvåking og evaluering, Seksuell og reproduktiv helse, Helse for mødre, barn og unge, Humanitær datarapportering, Helseinformasjonsystemer, Flyktninger, Flyktninghelse

## Abstract

**Background:**

Reliable and rigorously collected sexual, reproductive, maternal, newborn, child, and adolescent health (SRMNCAH) data in humanitarian settings are often sparse and variable in quality across different humanitarian settings, and there is a lack of consensus about a core set of indicators that humanitarian actors including national health systems should report on. To address this gap in quality data, the World Health Organization (WHO) developed a core set of indicators for monitoring and evaluating SRMNCAH services and outcomes and assessed their feasibility in four countries, including the Democratic Republic of Congo (DRC) with the goal of aggregating information from global consultations and field-level assessments to reach consensus on a set of core SRMNCAH indicators among WHO partners.

**Methods:**

The feasibility assessment in the DRC focused on the following constructs: relevance/usefulness, feasibility of measurement, systems and resources, and ethical issues. The multi-methods assessment included five components; a desk review, key informant interviews, focus group discussions, facility assessments, and observational sessions.

**Results:**

The findings suggest that there is widespread support among stakeholders for developing a standardized core list of SRMNCAH indicators to be collected among all humanitarian actors in the DRC. There are numerous resources and data collection systems that could be leveraged, built upon, and improved to ensure the feasibility of collecting this proposed set of indicators. However, the data collection load requested from donors, the national government, international and UN agencies, and coordination/cluster systems must be better harmonized, standardized, and less burdensome.

**Conclusions:**

Despite stakeholder support in developing a core set of indicators, this would only be useful if it has the buy-in from the international community. Greater harmonization and coordination, alongside increased resource allocation, would improve data collection efforts and allow stakeholders to meet indicators’ reporting requirements.

## Plain Language Summary

In humanitarian settings, data collection is often unreliable and not standardized—especially with regards to sexual, reproductive, maternal, newborn, child, and adolescent health (SRMNCAH). In order to address this gap in data quality, the World Health Organization proposed a list of core indicators to four countries, including the Democratic Republic of Congo (DRC). The goal was to use the findings from the DRC context to contribute to the determination of a core set of indicators. This study had two components: a feasibility assessment and a multi-methods assessment. The feasibility assessment in the DRC focused on the relevance of the proposed indicators, the feasibility of measurement, the resources in place, and ethical issues. The multi-methods assessment included a desk review, interviews with key informants, and focus groups and facility assessments. The findings showed that partners in the DRC supported developing a standard set of SRMCAH indicators that can be used among all humanitarian partners in the country. There are many systems in place that could be used or strengthened to improve data collection. However, the findings also showed that there must be better collaboration and coordination between the different partners in the DRC, as well as increased resources, to alleviate burden on frontline staff. In conclusion, though there is a desire to harmonize indicators, more input and resources are needed from the international community to aid in the standardization of data collection in order to meet local reporting requirements and ease burden on local staff.

## Résumé

**Contexte:**

Les données fiables et rigoureusement recueillies sur la santé sexuelle, reproductive, maternelle, néonatale, infantile et adolescente (SSRMNIA) dans les contextes humanitaires sont souvent rares et de qualité variable d'un contexte humanitaire à l'autre. Il n'y a pas de consensus sur un ensemble d'indicateurs de base sur lesquels les acteurs humanitaires, y compris les systèmes de santé nationaux, devraient se rapporter. Pour remédier à ce manque de données de qualité, l’Organisation mondiale de la santé (OMS) a établi un ensemble d’indicateurs de base pour le contrôle et l’évaluation des services et des résultats de la SSRMNIA et a évalué leur faisabilité dans quatre pays, y compris la République démocratique du Congo (RDC), dans le but d'agréger les informations provenant des consultations mondiales et des évaluations sur le terrain pour parvenir à un consensus sur un ensemble d'indicateurs de base de la SSRMNIA parmi les partenaires de l'OMS.

**Méthodes:**

L'évaluation de la faisabilité en RDC a porté sur les concepts suivants : pertinence/utilité, faisabilité de la mesure, systèmes et ressources, et questions d'éthique. L'évaluation multiméthodes comprenait cinq composantes : une étude documentaire, des entretiens avec des informateurs clés, des discussions de groupe, des évaluations d'installations et des séances d'observation.

**Résultats:**

Les résultats suggèrent qu'il y a un soutien généralisé parmi les acteurs concernés pour le développement d'une liste de base standardisée d'indicateurs pour la SSRMNIA à être recueilli parmi tous les acteurs humanitaires en RDC. Il existe de nombreuses ressources et plusieurs systèmes de collection de données qui pourraient être exploités, construits et améliorés afin d’assurer la faisabilité de la collection de l’ensemble des indicateurs proposés. Cependant, la charge de la collecte de données sollicitée auprès des donateurs, du gouvernement national, des agences internationales et de l'ONU, et les systèmes de coordination/clusters doivent être davantage harmonisés, standardisés, et moins fastidieux.

**Conclusions:**

Malgré le soutien des acteurs concernés à l'élaboration d'un ensemble d'indicateurs de base, celui-ci ne sera utile que s'il reçoit l’appui de la communauté internationale. Une harmonisation et une coordination accrue, ainsi qu'une meilleure allocation des ressources qui amélioreraient les efforts de collection de données et permettraient aux acteurs de satisfaire aux exigences de déclaration des indicateurs.

## Abstract (no)

**Bakgrunn:**

Pålitelige og strengt innsamlede data om seksuell, reproduktiv, mødre-, nyfødt-, barn- og ungdomshelse (SRMNCAH) i humanitære miljøer er ofte sparsomme og varierende i kvalitet på tvers av ulike humanitære miljøer, og det er mangel på konsensus om et kjernesett av indikatorer som humanitære aktører inkludert nasjonale helsesystemer bør rapportere om. For å løse dette gapet i kvalitetsdata utviklet Verdens helseorganisasjon (WHO) et kjernesett med indikatorer for overvåking og evaluering av SRMNCAH-tjenester og resultater og vurderte deres gjennomførbarhet i fire land, inkludert Den demokratiske republikken Kongo (DRC) med mål om samle informasjon fra globale konsultasjoner og feltnivåvurderinger for å oppnå konsensus om et sett med kjerne SRMNCAH-indikatorer blant WHO-partnere.

**Metoder:**

Gjennomførbarhetsvurderingen i DRC fokuserte på følgende konstruksjoner: relevans/nytte, gjennomførbarhet av måling, systemer og ressurser, og etiske spørsmål. Fler metodevurderingen inkluderte fem komponenter; en skrivebords anmeldelse, nøkkelinformantintervjuer, fokusgruppediskusjoner, fasilitetsvurderinger og observasjonssesjoner.

**Resultater:**

Funnene tyder på at det er bred støtte blant interessenter for å utvikle en standardisert kjerneliste over SRMNCAH-indikatorer som skal samles inn blant alle humanitære aktører i DRC. Det er mange ressurser og datainnsamlingssystemer som kan utnyttes, bygges på og forbedres for å sikre muligheten for å samle inn dette foreslåtte settet med indikatorer. Imidlertid må datainnsamlingsmengden som etterspørres fra givere, den nasjonale regjeringen, internasjonale og FN-byråer og koordinerings-/klyngesystemer være bedre harmonisert, standardisert og mindre tyngende.

**Konklusjoner:**

Til tross for interessentstøtte i å utvikle et kjernesett med indikatorer, vil dette bare være nyttig hvis det har støtte fra det internasjonale samfunnet. Større harmonisering og koordinering, sammen med økt ressursallokering, vil forbedre datainnsamlingsinnsatsen og tillate interessenter å møte indikatorenes rapporteringskrav.

## Background

### The humanitarian crisis in the Democratic Republic of Congo

The Democratic Republic of Congo (DRC) remains one of the most complex and long-standing humanitarian crises in the world [1]. According to the United Nations High Commissioner for Refugees (UNHCR), waves of political unrest have resulted in 5.01 million internally displaced persons (IDP) between October 2017 and September 2019 [1]. Tanganyika in the southeast, as well as the Kasaï provinces, have also faced war and unrest in recent years [2]. The origins of the unrest seen in the DRC today stem from the massive refugee crisis from the 1994 genocide in Rwanda [3]. The COVID-19 pandemic has only exacerbated the situation. Along with the Ebola epidemic, which the DRC has been fighting since 2018, there is significant strain on the health care system [4].

Women and children are particularly vulnerable in volatile environments when safety is not guaranteed [5]. In conflict-affected settings, sexual, reproductive, maternal, neonatal, child, and adolescent health (SRMNCAH) needs increase while available services and resources decrease [6]. Evidence suggests that women in these settings are at higher risk of death and disability due to pregnancy-related causes [7], have more difficulty with accessing sexual and reproductive health and abortion services [7], and experience higher rates of unintended and unwanted pregnancies, gender-based violence [8], and sexually transmitted infections, including HIV [9].

### SRMNCAH data collection and indicator reporting

Reliable and timely data have a direct bearing on a country’s capability to design and implement programmes effectively. Rigorous and regular data collection, storage, and analysis would contribute to measurable assessments of the objectives, goals and purpose of initiatives in terms of process, impact and intended outcome, both in organisational process and financial accountability [10–13]. Furthermore, timely and rigorous collection, aggregation, and use of SRMNCAH data for services and outcomes evaluation in humanitarian settings is an important component of accountability and transparency [12, 13]. This, in turn, ensures accountability to the beneficiaries—and that programs delivered by NGO partners and/or funded by donors or government improved lives through competent, equitable and dignified approaches. The key driver in accountability is ensuring that the affected population’s needs are met with dignity and humanity, by having transparent and clear SRHMNCAH data to help understand program outcomes and to continuously evaluate and improve [12]. Furthermore, these data collection and indicators’ reporting mechanisms would ensure that SRMNCAH service provision are meeting the global standard of care—the state/government becomes accountable by providing appropriate SRMNCAH care to its population [10, 14].

This type of data collection system, when implemented correctly in a reliable and timely manner, could allow governments and implementing agencies to accurately monitor and assess current services and outcomes in humanitarian settings as well as evaluate the impact of programs and budget allocations [10]. However, reliable and rigorously collected SRMNCAH data in humanitarian settings are often sparse and variable in quality [10]. A 2012–2014 global assessment found significant gaps in information about SRHMNCAH in refugee and displacement settings, irrespective of region or stage of emergency [15]. A recent study estimated the score of the quality of the DRC National Health Information System (NHIS) data at 40% [16].

### SRMNCAH data collection and indicator reporting in the DRC

In 2014, to improve the collection, storage and accessibility of data, the Ministry of Health (MoH) of the DRC introduced the District Health Information System 2 (DHIS2), an electronic, web-based management information system, which aids with the collection, presentation, analysis and validation of health information data [17]. Assessments of the DHIS2 in the DRC indicate improved quality and availability of health statistics and health data, which has helped to inform action in the health system [18]. However, the progress of DHIS2 was limited due to poor internet connectivity, lack of skills from data managers and other stakeholders, as well as a lack of suitable computers [19]. Other challenges included the constant closing of health facilities due to political instability and a very high turnover rate of health zone data managers [19]. As a result, significant gaps in quality SRMCNAH data remain, inhibiting key stakeholders from developing and implementing timely evidence-based interventions [10].

### Aims and objectives

In light of the above, the WHO in close coordination with local, regional, and global partners agreed to test the feasibility of a candidate set of SRMNCAH indicators for humanitarian settings at the field level in four countries experiencing different types of humanitarian crises, including the DRC, to determine feasibility, relevance, and acceptability. It should be noted that this framework was developed based on the 2018 Interagency Field Manual for Reproductive Health in Crisis (IAFM), relevant sphere indicators, WHO 100 core set of indicators, Every Woman Every Child strategy indicators and other technical and normative guidelines,^1^,^2^ as well as a systematic review of existing indicators^3^ conducted in 2018. The WHO began the process of developing a common core framework for monitoring SRMNCAH programs (services and outcomes) in humanitarian settings with donors, humanitarian partners from United Nations (UN) agencies, representatives from international non-governmental organizations (NGOs), and representatives from various WHO regional offices. In this article, we discuss the results of this assessment in the DRC. The assessment took place in Kinshasa (the capital of DRC), and the provinces of Kasaï, Kasaï Central, Kasaï Oriental.

By assessing feasibility, we aimed to explore the potential impact of the intended data collection and analysis, whether or not national and non-governmental monitoring and evaluation systems have the needed resources to collect SRMNCAH indicators, and the ability of the system to adhere to ethical practices and safeguard clients’ confidentiality and privacy [20]. The results of DRC’s country-level assessment will be eventually aggregated with the results from Afghanistan, Bangladesh, and Jordan’s field-level assessments in order to reach a global consensus on a minimum set of core SRMNCAH indicators for services and outcomes evaluation in humanitarian settings among donor agencies, United Nations (UN) agencies, and international non-governmental organizations (NGOs) working in humanitarian settings.

## Methods

### Study design

**Fig. 1:**
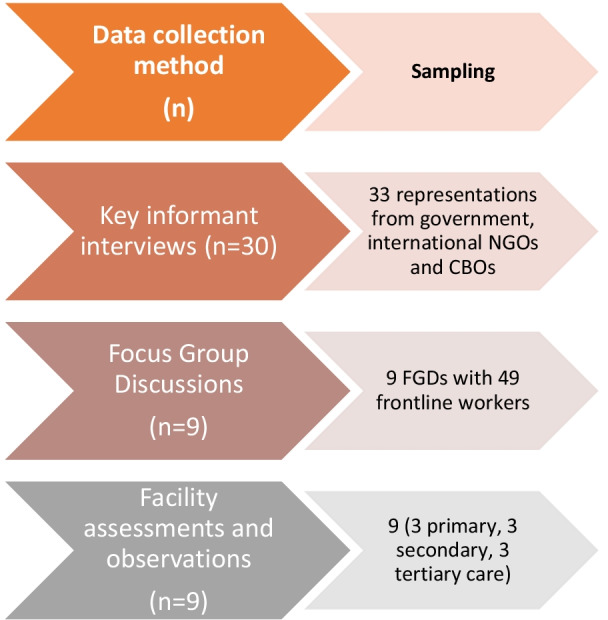
Data collection for Phase II. *NGO* non-governmental organization, *CBO* community based organization

This multi-methods assessment consisted of five main components: (1) a desk review of published articles and reports as well as internal documents (in English and French); (2) key informant interviews (KIIs) with representatives from government entities, health zone directorates, international and national NGOs, and staff members from health care clinics and hospitals; (3) facility assessments in primary and secondary facilities in the three Kasaï provinces that provide services to internally displaced and refugee populations; (4) observation sessions focused on the logistical, ethical, and privacy practices associated with data collection and storage at select facilities; and (5) focus group discussions (FGD) with frontline workers at primary, secondary, and tertiary health facilities (see Fig. 1).^4^

### Sampling

For the KIIs, we compiled a list of key individuals from government entities, international and national organizations that worked in the field of SRMNCAH and data collection at the different provincial levels in Kinshasa, the capital of DRC, and in the three Kasaï provinces. We interviewed key informants (KIs) individually or in small groups in the four study sites. For the FGDs and facility assessments, a list of facilities was compiled for each of the Kasaï provinces that provided SRMNCAH services to internally displaced and refugee populations (primary, secondary, and tertiary facilities). Each facility was informed, and the WHO country office in DRC facilitated the needed authorization prior to the evaluation. The facilities that were selected were identified as the largest subsidized providers for SRMNCAH services for internally displaced refugee populations in their respective provinces. Informed consent was obtained from each participant.

### Characteristics of study participants

KIIs took place between December 2019 and February 2020. We conducted **30 KIIs** with **33 representatives** from government entities, health zone directorates, international and national non-governmental organizations, and staff members from health care clinics and hospitals. We conducted **nine facility assessments** at primary, secondary, and tertiary facilities that provide services to refugees in the three provinces in the Kasaï region; observation sessions focused on the logistical, ethical, and privacy practices associated with data collection and storage at select facilities. Finally, we conducted **nine FGDs** with **49 frontline workers** at primary, secondary, and tertiary health centers (see Fig. 1).

### Desk review

The project was initiated with a comprehensive review of peer-reviewed literature, existing published and unpublished data, including institutional and donor reports that focused on SRMNCAH indicators’ reporting and analysis in DRC (specifically in the Kasaï region); coupled with an in-depth examination of the national SRMNCAH indicators’ list that organizations are required to report against on the DHIS2. Representatives from both governmental and non-governmental organizations in the DRC provided the study team with a set of key documents after each in-depth interview.

### Key informant interviews (n = 30, 33 total participants)

Using a semi-structured interview guide developed specifically for the overarching study, we focused on KIs’ perceptions and attitudes towards: SRMNCAH issues in the DRC (specifically in the Kasaï region), SRMNCAH service provision for refugee and internally displaced populations, current reporting practices on SRMNCAH indicators, and the feasibility of reporting on the candidate set of core SRHMNAH indicators. We also explored stakeholders’ perceptions and attitudes of current challenges in documenting and resources needed to successfully report against these indicators. We further explored the necessary buy-in needed among donor, governmental, and non-governmental agencies to enable the success of this effort.

### Facility assessments (n = 9)

We conducted nine facility assessments at primary, secondary and tertiary health facilities in the Kasaï provinces (three in each selected province). These assessments aimed to determine the nature and extent of SRMNCAH services offered, the ways in which patient information was collected, logged, stored, and safeguarded, and the types of human and technological resources used in data capture.

### Facility observation sessions (n = 9)

In conjunction with the facility assessments, observational sessions were also carried out in all nine facilities. These observational sessions aimed to assess existing resources currently being employed to collect data and additional resources needed to collect additional needed data for the core set of SRMNCAH indicators.

### Focus group discussions (n = 9; 49 total participants)

Nine FGDs were conducted with 49 frontline workers from nine clinics and hospitals across the Kasaï region. Participants provided verbal consent at the beginning of each FGD, which lasted an average of 90–120 min and took place in French. With consent, we audio-recorded all nine FGDs, debriefed as a team after each discussion, and wrote analytic memos to capture group dynamics and identify early themes.

### Analytic approach

An iterative, multi-phased approach was employed to analyze the data, such that analysis occurred simultaneously with data collection [21, 22]. All authors took part in the analysis, which also comprised of formal memos after each encounter, allowing for continuous identification of emerging themes and patterns from the KIIs and FGDs using both inductive and deductive techniques. We used a priori (pre-determined) codes based on the study aims and questions to analyze the content and themes from all the KIIs and FGDs. These findings were then combined with the results from the facility assessments, further complimented by validation discussions with KIs to ensure that data drove our analysis. NVivo was used to manage our data, which included transcripts, notes, and memos. We entered and analyzed the listed available indicator responses by organization in a password-protected Microsoft Excel file in a secure electronic database. The final recommendations were informed by feedback from WHO. The analysis focused on four core elements: (1) feasibility of collecting the proposed core set of SRMNCAH indicators, (2) relevance and usefulness of SRMNCAH data management mechanisms; (3) available existing resources and systems for national and humanitarian SRMNCAH data collection; and (4) ethical considerations of collecting and storing data.

### Research ethics

The Research Project Review Panel (RP2) of the WHO’s Department of Sexual and Reproductive Health reviewed and approved this study. We obtained authorization to conduct this study from the WHO’s Country Office in DRC and the DRC Ministry of Health (MoH). Additionally, we also obtained ethical approval to conduct this study from the National Ethical Committee of DRC (n°144/CNES/BN/PMMF/2018 on 2/11/2019). Furthermore, we received authorization from the MoH at the national and provincial levels. The Social Sciences and Humanities Research Ethics Board of the University of Ottawa provided ethical approval (Protocol number: S-08-18-1029).

## Findings

The results of this assessment focused on the four core elements highlighted in the study objectives: (1) feasibility of collecting the proposed core set of SRMNCAH indicators, (2) relevance and usefulness of SRMNCAH data management mechanisms; (3) available existing resources and systems for national and humanitarian SRMNCAH data collection; and (4) ethical considerations. We first start by outlining the feasibility of collecting the proposed SRMNCAH indicators, then we move to describing the current and potential advantages as well as challenges with SRMNCAH data capturing, followed by outlining the available data collections systems for the proposed indicators by the different humanitarian agencies. The findings section will conclude by discussing the different enforced measures in DRC to protect data privacy and confidentiality among the different implementing agencies.

### (1) Feasibility

The findings of the study show that of the proposed indicators, 70% were deemed feasible and relevant in the DRC, in domains including contraception (75%), maternal health (76%), newborn health (88%), child health (60%), sexual and gender-based violence (43%), prevention of mother-to-child transmission (100%), and sexually transmitted and blood-borne infections (100%) (see Table 1). Of the indicators deemed feasible and relevant, 73% are currently being collected (see Tables 1 and 2). The findings also show that some proposed indicators were deemed unfeasible and irrelevant, such as adolescent health-related indicators (33%) and comprehensive abortion care (40%). Table 2 gives an overview of the included and excluded list of indicators, the reported percentage of agencies who are currently collecting the indicator, site of data collection, their respective facilitators and barriers for routine data collection, any necessary modifications, and resources for routine data collection.

**Table 1:** Summary and percentage of the indicators by domain that were perceived relevant and feasible to the Congolese context, by number and percentage respectively

	Number of indicators by domain (n)	Number of indicators relevant to the Congolese context (n)	% of indicators that are feasible (%)
Contraception	4	3	75
Comprehensive abortion care	5	2	40
Maternal health	17	13	76
Newborn health	16	14	88
Child health	10	6	60
Adolescent HEALTH	6	2	33
Sexual and gender-based violence	7	3	43
HIV	3	3	100
Prevention from mother to child	4	4	100
Sexually transmitted infections (STIs) and reproductive tract infections (RTIs)	1	1	100
Total	73	51	70

**Table 2:** Summary findings of the feasibility of collecting the following proposed SRMNCAH indicators in the humanitarian context of the Democratic Republic of the Congo

No.	Indicator name	Overall % of agencies reporting	Overall % of agencies reporting	Overall % of agencies reporting	Place of collection	Facilitators to routine collection	Barriers to routine collection	Necessary modifications	Resources needed for routine collection	Exclude/include
		Kasai	Kasai Central	Kasai Oriental						
*Contraception*										
1.1	Number of clients initiating contraception	100%	56%	89%		National System for Health Information paper registers DHIS2	Low and unreliable availability of commodities Need for different contraception modalities	*# of clients accepting a new modern contraceptive method, by method* Should be coupled with indicators covering use, discontinuation and trained staff on provision of modern contraceptive methods	Training for all primary care providers on the different modalities of contraception	Include
1.2	Number of clients receiving emergency contraception	100%	44%	78%		National System for Health Information paper registers DHIS2	Low and unreliable availability of commodities Need for different contraception modalities	Should be coupled with an indicator tracking the number of resources available in clinic and indicator on number trained staff on provision of modern contraceptive methods	Training on the new IAFM guidelines Train primary care providers on the different contraceptive modalities that can be used for EC	Include
1.3	Percentage of clients adopting modern contraceptive method after delivery	100%	56%	89%		National System for Health Information paper registers DHIS2	Low and unreliable availability of commodities Need for different contraception modalities	N/A	N/A	Include
1.4	Percentage of clients adopting modern contraceptive method after abortion	100%	11%	78%		N/A	Legal status of abortion in DRC Service not provided Potential risk for patient and primary care provider	N/A	N/A	Exclude
*Comprehensive abortion care*										
2.1	Number of clients requesting an abortion	0%	0%	11%		N/A	Legal status of abortion in DRC Potential risk for patient and primary care provider Insufficient data encryption	N/A	N/A	Exclude
2.2	Number of clients receiving an abortion referral	38%	0%	44%		N/A	Legal status of abortion in DRC Potential risk for patient and primary care provider Insufficient data encryption	N/A	N/A	Exclude
2.3	Number of clients receiving an induced abortion	38%	0%	44%		N/A	Potential risk for patient and primary care provider Induced abortions are illegal in the DRC except in limited situations according to the Maputo Protocol; however, this exception has yet to be written into national law. Though care can be provided to clients presenting with having self-induced an abortion, due to its rarity and potential repercussions, this indicator is not collected Insufficient data encryption	N/A	N/A	Exclude
2.4	Number of clients presenting for post-abortion care (PAC)	88%	0%	89%		National System for Health Information paper registers DHIS2	Only secondary and tertiary health facilities are equipped and trained to provide comprehensive PAC	N/A	Training on the new IAFM guidelines DHIS2 create anonymity for this indicator (code) Service mapping of abortion providers who provide abortion services to the fullest extent of the law	Include
2.5	Number of clients receiving PAC	100%	22%	78%		National System for Health Information paper registers DHIS2	Only secondary and tertiary health facilities are equipped and trained to provide comprehensive PAC	N/A	Training on the new IAFM guidelines DHIS2 create anonymity for this indicator (code) Service mapping of abortion providers who provide abortion services to the fullest extent of the law	Include
*Maternal health*										
3.1	Number of maternal deaths	100%	44%	78%		National System for Health Information paper registers DHIS2	Weak infrastructures around national registries Perceived risk of sanctions on health facilities if a maternal death is reported	*# of maternal deaths in the facility, by cause of death* Should be disaggregated by cause and coupled with indicators that capture maternal death in the community	Increased transparency in auditing practices surrounding maternal death Resources will need to be developed and implemented at the community level to capture the indicators for the maternal deaths in the community	Include
3.2	Number of maternal deaths, disaggregated	38%	44%	44%		National System for Health Information paper registers DHIS2	Weak infrastructures around national registries Perceived risk of sanctions on health facilities if a maternal death is reported	*# of maternal deaths*^*1*^*, disaggregated by age (less than 15 years; between 15 and 19 and* equal *or greater than 19 years)*	Training for data collectors on the different causes of maternal death and how to encode for each Develop detailed manuals for frontline workers and data collectors Training and capacity building for community health workers Increased transparency in auditing practices surrounding maternal deaths	Include
3.3	Percentage of maternal death reviews	75%	11%	89%		National System for Health Information paper registers DHIS2	Weak infrastructures around national registries Perceived risk of sanctions on health facilities if a maternal death is reported	*# of maternal deaths in the facility that were audited and reviewed* French wording should be changed to “revue” as this is how it is reported in DHIS2	Training and capacity building for staff to review on maternal death cases Training and capacity building for community health workers Increased transparency in auditing practices surrounding maternal deaths	Include
3.4	Number of clients receiving antenatal care (ANC)	100%	44%	67%		National System for Health Information paper registers DHIS2	Weak infrastructures around national registries	N/A	Accessibility to commodities and supplies	Include
3.5	Number of deliveries	100%	44%	89%		National System for Health Information paper registers DHIS2	Weak infrastructures around national registries	*# of clients delivering in facility, including both live and stillbirths* Should be coupled with indicators to capture births occurring in the community	Frontline workers will need training on reporting on stillbirths Leveraging systems to capture stillbirths for community births (UNICEF) Training and capacity building for community health workers	Include
3.6	Number of deliveries, disaggregated	63%	22%	78%		National System for Health Information paper registers DHIS2	Weak infrastructures around national registries	*# of clients delivering in facility, including both live and stillbirths, disaggregated by age (less than 15 years; between 15–19 and equal or greater than 19 years)*	Frontline workers will need training on reporting on stillbirths Leveraging systems to capture stillbirths for community births (UNICEF) Training and capacity building for community health workers	Include
3.7	Number of clients receiving post-natal care (PNC)	88%	22%	78%		National System for Health Information paper registers DHIS2	Weak infrastructures around national registries	N/A	N/A	Include
3.8	Number of caesarean section deliveries	88%	11%	67%		National System for Health Information paper registers DHIS2	Weak infrastructures around national registries	Coupled with an indicator on the number of referrals for cesareans	Might not be useful outside of hospital setting	Include
3.9	Availability of PAC	100%	22%	67%		N/A	Service mapping exercise at the provincial level	N/A	N/A	Exclude
3.10	Availability of basic emergency obstetric care (BEmOC)	88%	33%	89%		N/A	Service mapping exercise	N/A	N/A	Exclude
3.11	Availability of comprehensive emergency obstetric care (CEmOC)	88%	22%	78%		N/A	Service mapping exercise	N/A	N/A	Exclude
3.12	Availability of skilled personnel	88%	22%	67%		N/A	Service mapping exercise	N/A	N/A	Exclude
3.13	Number of antenatal care clients with tetanus vaccination	100%	33%	89%		National System for Health Information paper registers DHIS2	Weak infrastructures around national registries	N/A	N/A	Include
3.14	Number of ANC clients receiving preventive therapy for malaria	88%	44%	89%		National System for Health Information paper registers DHIS2	Weak infrastructures around national registries	N/A	N/A	Include
3.15	Number of ANC clients receiving syphilis screening	50%	22%	33%		National System for Health Information paper registers DHIS2	Weak infrastructures around national registries Shortage/absence of diagnostic screening tests for Syphilis	N/A	N/A	Include
3.16	Number of ANC clients receiving urinary tract infection screening or treatment	88%	33%	67%		National System for Health Information paper registers DHIS2	Weak infrastructures around national registries	N/A	N/A	Include
3.17	Number of clients with identified maternal morbidities during post-natal care (PNC)	75%	33%	78%		National System for Health Information paper registers DHIS2	Weak infrastructures around national registries	N/A	N/A	Include
*Newborn health*										
4.1	Number of neonatal deaths	75%	44%	67%		National System for Health Information paper registers DHIS2	Perceived risk of sanctions on health facilities if a neonatal death is reported	*# of neonatal deaths (0–28) at the facility level* Should be coupled with an indicator tracking neonatal death within the community	Increased transparency in auditing practices surrounding neonatal death audits Training and capacity building for community health workers	Include
4.2	Number of stillbirths	75%	44%	67%		National System for Health Information paper registers DHIS2	Perceived risk of sanctions on health facilities if a neonatal death is reported Infrastructural barriers; lack of roads, electricity, and reliable internet connection	N/A	N/A	Include
4.3	Number of babies born low birth weight	75%	44%	78%		National System for Health Information paper registers DHIS2	Infrastructural barriers; lack of roads, electricity, and reliable internet connection	Should be coupled with an indicator tracking malnutrition among pregnant women	N/A	Include
4.4	Number of small and sick newborns receiving care	50%	22%	78%		National System for Health Information paper registers DHIS2	Infrastructural barriers; lack of roads, electricity, and reliable internet connection	N/A	N/A	Include
4.5	Number of newborns receiving post-natal care	63%	22%	67%		National System for Health Information paper registers DHIS2	Infrastructural barriers; lack of roads, electricity, and reliable internet connection	*# of newborns receiving post-natal care within 7–10 days*	N/A	Include
4.6	Availability of KMC	88%	22%	78%		N/A	Service mapping exercise	N/A	N/A	Exclude
4.7	Availability of neonatal resuscitation	75%	33%	78%		N/A	Service mapping exercise	N/A	N/A	Exclude
4.8	Number of neonatal deaths, disaggregated	63%	11%	67%		National System for Health Information paper registers DHIS2	Perceived risk of sanctions on health facilities if a neonatal death is reported	N/A	When reviewing data and making recommendations for health programs, consider the disaggregated indicator Leverage systems that capture newborn death and review these cases for cause of death Training and capacity building for of primary care providers	Include
4.9	Percentage of perinatal death reviews	25%	22%	67%		National System for Health Information paper registers DHIS2	Perceived risk of sanctions on health facilities if a neonatal death is reported	French wording should be changed to “revue” as this is how it is reported in DHIS2	In urban settings, national systems would need to be established to capture newborn death and review these cases for cause of death Training and capacity building of primary care providers Will need to be added to the DHIS2	Include
4.10	Number of newborns receiving Hepatitis B vaccine	0%	0%	22%		National System for Health Information paper registers DHIS2	Absence/shortages of the diagnostic screening test for Hepatitis B	N/A	Will need to be added to the DHIS2 Extensive training and capacity building will need to be implementing for scale-up at the national level	Include
4.11	Number of newborns initiating breastfeeding early	75%	33%	56%		National System for Health Information paper registers DHIS2	Infrastructural barriers; lack of roads, electricity, and reliable internet connection	N/A	N/A	Include
4.12	Number of infants weighed at birth	75%	11%	56%		National System for Health Information paper registers DHIS2	Infrastructural barriers; lack of roads, electricity, and reliable internet connection	*# of newborns weighed at delivery*	N/A	Include
4.13	Number of babies registered	63%	22%	67%		National System for Health Information paper registers DHIS2	Civil registry is unreliable	Distinguish between registration at the facility level and at the state level	Civil registries to be integrated into hospitals and clinics Significant training and resources will need be implemented	Include
4.14	Number of newborns receiving treatment for possible severe bacterial infection (PSBI)	63%	0%	56%		National System for Health Information paper registers DHIS2	Infrastructural barriers; lack of roads, electricity, and reliable internet connection	N/A	Significant training and resources will need be implemented	Include
4.15	Number of newborns admitted	25%	11%	44%		National System for Health Information paper registers DHIS2	Infrastructural barriers; lack of roads, electricity, and reliable internet connection	N/A	N/A	Include
4.16	Number of newborns with morbidities identified during PNC	38%	0%	44%		National System for Health Information paper registers DHIS2	Infrastructural barriers; lack of roads, electricity, and reliable internet connection	N/A	Resources for primary care providers on the definitions for morbidity type Will need to be added to the DHIS2 Extensive training and capacity building of primary care providers	Include
*Child health*										
5.1	Number of deaths of children under 5	88%	0%	44%		National System for Health Information paper registers DHIS2	Lack of integration of child health into routine service delivery as a category of its own	N/A	Training and outreach programs will need to be implemented at the health facility and community level Child health indicators to be integrated into routine service delivery as a specific area of its own, including for data collection	Include
5.2	Under 5 mortality rate	25%	0%	33%		N/A	Population-level indicator with impractical denominator	N/A	N/A	Exclude
5.3	Percentage of children under 5 with suspected pneumonia taken to appropriate health facility	50%	14%	33%		N/A	Population-level indicator with impractical denominator	N/A	N/A	Exclude
5.4	Coverage of diarrhea treatment	50%	14%	44%		National System for Health Information paper registers DHIS2	Lack of integration of child health into routine service delivery as a category of its own	N/A	Child health indicators to be integrated into routine service delivery as a specific area of its own, including for data collection	Include
5.5	Percentage of children under 5 who are wasted	38%	0%	44%		N/A	Low burden of disease, population-level indicator with impractical denominator	N/A	N/A	Exclude
5.6	Percentage of children under 5 who are registered	25%	0%	56%		N/A	The denominator of this indicator is not feasible since population level metrics are often unreliable in the DRC	N/A	N/A	Exclude
5.7	Number of children presenting with fever tested for malaria in endemic settings	50%	14%	56%		National System for Health Information paper registers DHIS2	Reported solely for donors; not integrated into national HIS	*# of children under 5 who have been administered outpatient malaria tests, by age*	Child health indicators to be integrated into routine service delivery as a specific area of its own, including for data collection	Include
5.8	Number of confirmed cases of malaria in endemic settings	50%	14%	56%		National System for Health Information paper registers DHIS2	Reported solely for donors; not integrated into national HIS	N/A	Child health indicators to be integrated into routine service delivery as a specific area of its own, including for data collection	Include
5.9	Percentage of confirmed malaria cases treated	50%	14%	56%		National System for Health Information paper registers DHIS2	Reported solely for donors; not integrated into national HIS	N/A	Child health indicators to be integrated into routine service delivery as a specific area of its own, including for data collection	Include
5.10	Coverage of DP3	50%	14%	56%		National System for Health Information paper registers DHIS2	Lack of integration of child health into routine service delivery as a category of its own	N/A	Child health indicators to be integrated into routine service delivery as a specific area of its own, including for data collection	Include
*Adolescent health*										
6.1	Adolescent birth rate	25%	14%	33%		N/A	Population-level indicator with impractical denominator	N/A	N/A	Exclude
6.2	Sexual violence against children	63%	25%	33%		N/A	Lack of integration of adolescent health services tailored to this population’s unique needs	N/A	N/A	Exclude
6.3	Adolescent mortality rate	0%	0%	11%		N/A	Population-level indicator with impractical denominator	N/A	N/A	Exclude
6.4	Percentage of adolescents living with HIV who are currently receiving antiretroviral therapy, disaggregated	Could be collected				Organizations in the DRC do not actively collect disaggregated data yet the information could be extracted from the HIS	Lack of integration of adolescent health services tailored to this population's unique needs	*# of adolescents living with HIV who are currently receiving antiretroviral therapy, disaggregated by age (less than 15 years; between 15 and 19 and equal or greater than 19 years)* Removal of the denominator	Significant training and capacity development for frontline staff on adolescent health indicator reporting Will need to be added to the DHIS2	Include
6.5	Immunization coverage rate	Could be collected				Organizations in the DRC do not actively collect disaggregated data yet the information could be extracted from the HIS	Lack of integration of adolescent health services tailored to this population's unique needs	*# of adolescents receiving the nationally mandated immunization, disaggregated by age (less than 15 years; between 15–19 and equal or greater than 19 years)* Removal of the denominator	Significant training and capacity development for frontline staff on adolescent health indicator reporting Will need to be added to the DHIS2	Include
6.6	Suicide rate, disaggregated	0%	0%	22%		N/A	Population-level indicator with impractical denominator Low burden of disease	N/A	N/A	Exclude
*Sexual and gender-based violence*										
7.1	Number of rape survivors	88%	50%	78%		National System for Health Information paper registers DHIS2	Current indicators do not capture the cultural and community interventions surrounding SGBV	N/A	An anonymous code should be assigned to each survivor to avoid duplication of data	Include
7.2	Percentage of health facilities with clinical management of rape services	63%	43%	67%		N/A	Service mapping exercise	N/A	N/A	Exclude
7.3	Percentage of rape survivors receiving HIV post-exposure prophylaxis	63%	29%	67%		National System for Health Information paper registers DHIS2	Current indicators do not capture the cultural and community interventions surrounding SGBV	Should be coupled with indicators capturing referrals and availability of supplies	Significant training for the entire health care team Training on the new IAFM Inclusion of community leaders Inclusion of judicial system Training and outreach for community leaders Changes in the DHIS2 will need to be made to avoid duplicity in the data reporting Training in clinical management of HIV Increased availability/supply of PEP kits Coordination with the Justice System	Include
7.4	Percentage of rape survivors receiving emergency contraception	88%	38%	67%		National System for Health Information paper registers DHIS2	Current indicators do not capture the cultural and community interventions surrounding SGBV	Should be coupled with indicators capturing referrals and availability of supplies	Significant training for the entire health care team Training on the new IAFM Training and outreach for community leaders Inclusion of judicial system Changes in the DHIS2 will need to be made to avoid duplicity in the data reporting Coordination with the Justice System	Include
7.5	Number of rape survivors requesting abortion	13%	0%	11%		N/A	Though it is technically legal to receive an abortion due to rape according to the Maputo Protocol, this exception has yet to be written into national law. As such, this indicator is not collected	N/A	N/A	Exclude
7.6	Number of rape survivors receiving induced abortion care or referral	25%	0%	44%		N/A	Though it is technically legal to receive an abortion due to rape according to the Maputo Protocol, this exception has yet to be written into national law. Though care will be provided to clients presenting with abortion, due to its rarity and potential repercussions, this indicator is not collected	N/A	N/A	Exclude
7.7	Availability of intimate partner violence front line support (LIVES)	25%	13%	44%		N/A	Service is not routinely provided	N/A	N/A	Exclude
*HIV*										
8.1	Antiretroviral therapy coverage among people living with HIV, disaggregated	38%	38%	56%		National System for Health Information paper registers DHIS2	Supply and training shortages	N/A	Training in clinical management of HIV	Include
8.2	Percentage of exposed individuals receiving post-exposure prophylaxis	38%	38%	56%		National System for Health Information paper registers DHIS2	Supply and training shortages	N/A	Training in clinical management of HIV	Include
8.3	Percentage of donated blood units screened for HIV in quality assured manner	38%	13%	56%		National System for Health Information paper registers DHIS2	Supply and training shortages	N/A	Training in clinical management of HIV Might not be useful outside of hospital setting	Include
*Prevention of mother-to-child transmission*										
9.1	Percentage of antenatal care clients receiving syphilis screening and treatment	50%	25%	44%		National System for Health Information paper registers DHIS2	Shortage/absence of diagnostic screening tests for Syphilis	N/A	Training in clinical management of HIV Might not be useful outside of hospital setting	Include
9.2	Percentage of antenatal care clients offered testing for HIV	50%	25%	56%		National System for Health Information paper registers DHIS2	Supply and training shortages	*# of first-time antenatal care clients who received pre-testing counselling for HIV*	Training in clinical management of HIV	Include
9.3	Percentage of HIV-positive pregnant people receiving antiretroviral therapy	63%	25%	44%		National System for Health Information paper registers DHIS2	Supply and training shortages	N/A	Training in clinical management of HIV	Include
9.4	Percentage of all deliveries to HIV-positive mothers receiving antiretrovirals	50%	25%	44%		National System for Health Information paper registers DHIS2	Supply and training shortages	*#of HIV-positive mothers who receive ART according to national protocol* Should be coupled with an indicator for the infant also receiving ART	Training in clinical management of HIV	Include
*Sexually transmitted infections (STIs) and reproductive tract infections (RTIs)*										
10.1	Percentage of STI/RTI cases managed	75%	15%	78%		National System for Health Information paper registers DHIS2	Infrastructural barriers; lack of roads, electricity, and reliable internet connection	*# of patients with STI/RTI accessing services who are diagnosed symptomatically, and counselled according to protocol* Distinguish between the number of cases and the number of cases “managed” STI and RTI cases need to be formulated as separate indicators	Training on how to identify and report on STI/RTIs Training on the clinical management of STI/RTI cases	Include

Italicized text refers to current description of indicator

The results show that overall, health facilities in the DRC have implemented the collection of indicators related to contraception and have recommended the inclusion of indicators 1.1 to 1.3 (see Table 2). Stakeholders and FGD participants overwhelmingly noted the regional and institutional variability of the availabilities of commodities and types of contraception, which at times hindered the feasibility of collection. Consistent with the findings from facility assessments, KI and FGD participants noted the selective collection of emergency contraception-related indicators in certain health facilities, which in some cases is limited to individuals that require the clinical management of rape. Due to the legality of abortion in the DRC, the post-abortion care indicator (1.4) was suggested for removal.

The findings show that a vast majority of agencies and health facilities included in this study do not collect indicators related to abortions—the facility assessments confirmed this finding. Though the legal framework in the DRC allows for abortions in select instances, potential repercussions from government officials and the rarity of these situations mean indicators suggesting elective abortions (2.1 to 2.3) are either not collected or they are concealed under other health care services. As stated by an FGD participant in Kasaï Oriental, “First of all, 2.1 which says, ‘number of clients requesting an abortion.’ We know that our deontology prohibits abortion, first of all because everyone has the right to life, and also outside of that, abortion is performed only for therapeutic reasons and when it is truly needed to save the life of the mother. Since these are rare cases, we do not collect these indicators.” Indicators related to post-abortion care (2.4 and 2.5) would be feasible to collect provided certain measures for anonymity.

Facilities collect the majority of the proposed maternal health indicators as part of routine data collection, including comprehensive pre-natal and post-natal consultations up to 42 weeks post-partum, as noted from the findings of facility assessments. KIs explained that this is primarily due to the significant investments the DRC government has made into this domain. Some indicators that have variable levels of collection, but are feasible, include maternal deaths (3.1 to 3.3) due to perceived risks of sanctions, as any maternal death registered triggers an audit on the health facility. A medical coordinator concisely stated, “… Sanctions, the technical platform, also play a role in newborn health.” Indicators that were not considered feasible include the availabilities of post-abortion care (PAC) (3.9), basic emergency obstetric care (BEmOC) (3.10), comprehensive emergency obstetric care (CEmOC) (3.11), and skilled personnel (3.12), as a result of service mapping exercises needed at the provincial level.

In the same vein as maternal health, most indicators on newborn health are collected during post-natal consultations. Indicators recommended for exclusion here revolve around the need for service mapping exercises, namely the availabilities of kangaroo mother care (KMC) (4.6) and neonatal resuscitation (4.7). Some variability exists in the feasibility of child health indicators, as the collection of these indicators has not yet been integrated into routine service delivery as its own domain. Though there is a lack of collection of these indicators, significant resources and training would allow for greater capabilities in this domain. Indicators that have been deemed unfeasible by participants at all levels include those with population-level denominators, namely the under-5 mortality rate (U5MR) (5.2), percentage of children under 5 with suspected pneumonia taken to an appropriate health facility (5.3), and percentage of children under 5 who are wasted (5.5).

Adolescent health is another domain that has not been integrated into routine service delivery, and as such, sees an even greater lack of data collection. Indicators that were deemed not feasible by KI and FGD participants include the adolescent birth rate (6.1), sexual violence against children (6.2), the adolescent mortality rate (6.3), and suicide rate (6.6)—primarily due to impractical population-level denominators and sociocultural barriers. Of all proposed indicators, percentage of adolescents living with HIV receiving antiretroviral therapy (6.4) remains feasible due to the existing infrastructure in place for HIV surveillance. Immunization coverage rate (6.5) could also be a feasible indicator with significant training and capacity development for frontline staff.

All participants noted existing gaps in the domain of sexual and gender-based violence. Significant resources would need to be provided to increase the feasibility of collecting these indicators across the country. Indicators that were considered feasible and relevant from facility assessments and FGD interviews include the number of rape survivors (7.1) provided they are anonymized, and rape survivors receiving HIV post-exposure prophylaxis (PEP) (7.3) and emergency contraception (7.4) provided there is  increased collaboration with government departments and community leaders. Indicators deemed unfeasible by FGD participants, KIs, and health facilities include the percentage of health facilities with clinical management of rape services (7.2) which would need a service mapping exercise, rape survivors requesting or receiving abortions (7.5 and 7.6) due to issues surrounding legality, and availability of LIVES^5^ (7.7) as this service is not routinely provided.

All of the indicators within the domains of HIV, prevention of mother-to-child transmission, and STI & RTI were deemed feasible and relevant. Though regional differences exist in the current collection of these indicators, KIs and FGD participants noted they would all be feasible following training on the clinical management of HIV, availability of resources and materials, capacity building of staff for service delivery, and training on how to identify and report STI/RTIs.

Many proposed barriers to feasibility exist at the facility level, where factors such as the availability of resources and training impede the ability of local providers at the health centre and general referral hospital level to collect indicators. Stakeholders note that continued armed conflict, looting, and infrastructure challenges continue to be barriers in the provision in care and data collection. The lack of both human and financial resources at the facility level plays a significant role in the collection of indicators, and in turn, the length of proposed indicators. Notably, participants stated that facility-based and contextually relevant data are more feasible to collect when compared to indicators that included a population-level denominator.

### (2) Relevance and usefulness of humanitarian SRMNCAH data management mechanisms

#### Perceived advantages with current and proposed SRMNCAH indicator reporting

The findings from our study indicate that KIs at the different levels of the health system were all in consensus that accurate and reliable SRMNCAH data provide opportunities for implementing evidence-based programming, improving service provision and the outcomes of its population, and ensuring that the service provision meets the global standard of care. Participants noted that SRMNCAH data collection allowed provinces to monitor and evaluate their progress in meeting their provincial health rate goals. As proudly stated by a KI, “[If] the objective is 20% and at times the health zone has reached up to 25%. The objective for reproductive health is 20%. As in we have to manage to cover 20% of the times between births…we are around 25%. We can see that we exceeded the objectives”. The majority of our participants also noted that the collection and reporting of SRMNCAH indicators’ purpose is to enable the MoH in their allocation of funds to the different health zones and to define and/or amend health priorities.

In general, stakeholders from all agencies and across the health system supported the general contours of the proposed core set of SRMNCAH indicators and noted that many of the proposed indicators are currently being collected through their centralized NHIS. A number of participants noted that the formation and use of DHIS2 allows for population-level reporting; however, given infrastructural and socio-cultural barriers, the reliability of these indicators is impugned. Even if indicators were not currently being collected, KIs and FGD participants could generally envision how to incorporate this information into facility-level data collection systems and/or extract from the data in DHIS2. Finally, a number of our participants noted the importance of ensuring that the set SRMNCAH indicator list mirror the set of SRMNCAH indicators selected by the MoH to avoid parallel systems, and in turn, ensure greater buy-in among key actors in the field.

#### Perceived disadvantages with current SRMNCAH indicator reporting

In contrast to KIs at the national and provincial level, health zone level participants were unable to identify the relevance and utility of SRMNCAH data outside from program evaluation requirements. As noted by many of our FGD participants, staff at health facilities were performing most of the labour with the least resources in addition to receiving little to no training on the purpose of SRMNCAH data collection exercises. Our findings also indicate that there are a number of disadvantages with capturing certain SRMNCAH indicators, specifically surrounding maternal and newborn health, and sexual and gender-based violence (SGBV). As noted by many of our stakeholders and FGD participants, a multitude of barriers contribute to the significant underreporting for maternal, newborn, abortion, and SGBV cases in the DRC. The findings suggest that the pressure arising from the politicization of SGBV, abortion, maternal, and newborn health indicators may lead certain facilities to underreport the number of cases.

#### Perceived gaps in the proposed SRMNCAH indicators

##### Indicators that should be removed from the core set of SRMNCAH indicators

Both KIs and frontline workers identified a number of the proposed SRMNCAH indicators as not relevant and/or useful for the humanitarian and developmental context of the DRC. Stakeholders from Kinshasa and across the Kasaï region believed a sub-set of indicators should be removed because: (1) the indicator relates to a service that is not (routinely) provided in the DRC context (i.e., number of children who are wasted, adolescent suicide and the LIVES intervention in cases of intimate partner violence); (2) national regulations and protocols restricting the provision of care and therefore the collection of information on the subject; and/or (3) the indicator would not have any practical or actionable applications (see Table 3).

**Table 3:** List of indicators that should be excluded according to our stakeholders, with primary rationale

	Indicator number and name	Rationale for exclusion
Contraception	1.4: Percentage of clients adopting modern contraception method after abortion	Service not provided Potential risk for patient and primary care provider^a^
Comprehensive abortion care	2.1: Number of clients requesting an abortion	Potential risk for patient and primary care provider Legal status of abortion in DRC Insufficient data encryption
	2.2: Number of clients receiving an abortion referral	Legal status of abortion in DRC Potential risk for patient and primary care provider Insufficient data encryption
	2.3: Number of clients receiving an induced abortion	Potential risk for patient and primary care provider Induced abortions are illegal in the DRC except in limited situations according to the Maputo Protocol; however, this exception has yet to be written into national law. Though care can be provided to clients presenting with having self-induced an abortion, due to its rarity and potential repercussions, this indicator is not collected Insufficient data encryption
Maternal health	3.9: Availability of PAC	Service mapping exercise at the provincial level^b^
	3.10: Availability of basic emergency obstetric care (BEmOC)	Service mapping exercise
	3.11: Availability of comprehensive emergency obstetric care (CEmOC)	Service mapping exercise
	3.12: Availability of skilled personal	Service mapping exercise
Newborn health	4.6: Availability of KMC	Service mapping exercise
	4.7: Availability of neonatal resuscitation	Service mapping exercise
Child health	5.2: Under 5 mortality rate	Population-level indicator with impractical denominator^c^
	5.3: Percentage of children under 5 with suspected pneumonia taken to appropriate health facility	Population-level indicator with impractical denominator
	5.5: Percentage of children under 5 who are wasted	Low burden of disease, population-level indicator with impractical denominator
	5.6: Percentage of children under 5 who are registered	The denominator of this indicator is not feasible since population level metrics are often unreliable in the DRC
Adolescent health	6.1: Adolescent birth date	Population-level indicator with impractical denominator
	6.2: Sexual violence against children	Information not actionable, population-level indicator with impractical denominator
	6.3: Adolescent mortality rate	Population-level indicator with impractical denominator
	6.6: Suicide rate, disaggregated	Population-level indicator with impractical denominator Low burden of disease^d^
Sexual and gender-based violence	7.2: Percentage of health facilities with clinical management of rape services	Service mapping exercise
	7.5: Number of rape survivors requesting abortion	Though it is technically legal to receive an abortion due to rape according to the Maputo Protocol, this exception has yet to be written into national law. As such, this indicator is not collected
	7.6: Number of rape survivors receiving induced abortion care or referral	Though it is technically legal to receive an abortion due to rape according to the Maputo Protocol, this exception has yet to be written into national law. Though care will be provided to clients presenting with abortion, due to its rarity and potential repercussions, this indicator is not collected
	7.7: Availability of intimate partner violence front line support (LIVES)	Service not routinely provided
HIV	No indicator indicated for exclusions in this section	
Prevention of mother-to-child transmission	No indicator indicated for exclusions in this section	
Sexually transmitted infections (STIs) and reproductive tract infections (RTIs)	No indicator indicated for exclusions in this section	

^a^According to the penal code of the Democratic Republic of Congo (DRC), abortion is prohibited without exception. Legal prohibition notwithstanding, it is generally accepted that the procedure can be performed to save a woman’s life; yet, women are rarely able to obtain safe abortion care on this basis. Despite DRC signatory to the Maputo Protocol, the vast majority of abortions occurring in the DRC are clandestine, and many of these are unsafe. The Maputo Protocol was signed by the DRC in March 2018 and was published into the national legal gazette, officially entering the treaty into force

^b^Service mapping exercise aims to examine what services and programs, and by whom are offered; the services and programs that other agencies within the same communities are offering. It seems to also examine the links between services and programs provided and utilized by the target population. Service mapping also aims to identify any service and/or programming gaps that may exist in the community that might need to be addressed

^c^Our stakeholders repeatedly invalidated population level data, due to weak infrastructure and national registries. Given the unreliability of population level data, indicators that include a denominator is impractical and should be reworded as a facility-based indicator

^d^Perceived low burden disease reported by the research study participants

All of our stakeholders in the DRC raised concerns regarding the inclusion of abortion-related indicators. Our key informants explained that due to cultural and regulatory barriers, the abortion-related indictors should be removed to safeguard health zones, health care providers and women seeking abortions. A number of our KIs explained that although the DRC acceded to the Maputo Protocol^6^ in 2006 and published this decision in the country's official journal in 2018, acceptance of comprehensive abortion care (CAC) has yet to occur due to the absence of amendments to the national penal code and institutional policies, compounded with socio-cultural norms. These participants believed that given the lack of adoption of the protocol into national regulations, health care providers and women seeking abortions are still at great risk of criminalization and community rejection, and health centres and provinces could be at further risk of sanctions.

##### Additional indicators that should be added to the core set of SRMNCAH indicators

Stakeholders in the DRC indicated that although the proposed list SRMNCAH indicators is comprehensive, there remains a need to include a number of indicators in the core list, including indicators relating to supply-chains, commodities/stockouts, and coordination. Our participants believed that the indicators on commodities/stockouts would enable the health facilities in ensuring commodity availability and for the national government and funders to accurately predict the volume of commodities needed in each health zone. Table 4 provides a list of additional indicators: the percentage of organizations currently collecting the proposed indicator, and the recommended resources and training needed for routine collection recommended by the study participants to be added to the core list of SRMNCAH indicators.

**Table 4:** List of indicators that were recommended to be added to the core list of indicators

	Indicator		Percentage of org. collecting this indicator (%)			Resources and training needed for routine collection
			Kasaï	Kasaï Central	Kasaï Oriental	
Contraception	1.1: Number of clients initiating contraception	*# of clients accepting a new modern contraceptive method, by method* Should be couple with indicators covering use, discontinuation and trained staff on provision of modern contraceptive methods	100%	56%	89%	Training for all primary care providers on the different modalities of contraception
	1.2: Number of clients receiving emergency contraception	Should be coupled with an indicator tracking the number of resources available in clinic and indicator on number trained staff on provision of modern contraceptive methods	100%	44%	78%	Training on the new IAFM guidelines^d^ Train primary care providers on the different contraceptive modalities that can be used for EC
	1.3: Percentage of clients adopting modern contraceptive method after delivery	N/A	100%	56%	89%	N/A
Maternal Health Abortion	2.4: Number of clients presenting for post-abortion care	N/A	88%	0%	89%	Training on the new IAFM guidelines DHIS2 create anonymity for this indicator^a^ (code) Service mapping^b^ of abortion providers who provide abortion services to the fullest extent of the law
	2.5: Number of clients receiving post-abortion care	N/A	100%	22%	78%	Training on the new IAFM guidelines DHIS2 create anonymity for this indicator^c^ (code) Service mapping of abortion providers who provide abortion services to the fullest extent of the law
	3.1: Number of maternal deaths	*# of maternal deaths in the facility, by cause of death* Should be disaggregated by cause and coupled with indicators that capture maternal death in the community	100%	44%	78%	Increased transparency in auditing practices surrounding maternal death Resources will need to be developed and implemented at the community level to capture the indicators for the maternal deaths in the community
	3.2: Number of maternal deaths, disaggregated	*# of maternal deaths*^*1*^*, disaggregated by age (less than 15 years; between 15 and 19 and* equal *or greater than 19 years)*	38%	44%	44%	Training for data collectors on the different causes of maternal death and how to encode for each Develop detailed manuals for frontline workers and data collectors Training and capacity building for community health workers Increased transparency in auditing practices surrounding maternal deaths
	3.3: Percentage of maternal death reviews	*# of maternal deaths in the facility that were audited and reviewed* French wording should be changed to “revue” as this is how it is reported in DHIS2	75%	11%	89%	Training and capacity building for staff to review on maternal death cases Training and capacity building for community health workers Increased transparency in auditing practices surrounding maternal deaths
	3.4: Number of clients receiving antenatal care	N/A	100%	44%	67%	Accessibility to commodities and supplies
	3.5: Number of deliveries	*# of clients delivering in facility, including both live and stillbirths* Should be coupled with indicators to capture births occurring in the community	100%	44%	89%	Frontline workers will need training on reporting on stillbirths Leveraging systems to capture stillbirths for community births (UNICEF) Training and capacity building for community health workers
	3.6: Number of deliveries, disaggregated	*# of clients delivering in facility, including both live and stillbirths, disaggregated by age (less than 15 years; between 15 and 19 and equal or greater than 19 years)*	63%	22%	78%	Frontline workers will need training on reporting on stillbirths Leveraging systems to capture stillbirths for community births (UNICEF) Training and capacity building for community health workers
	3.7: Number of clients receiving post-natal care	N/A	88%	22%	78%	N/A
	3.8: Number of caesarean section deliveries	Coupled with an indicator on the number of referrals for cesarians	88%	11%	67%	Might not be useful outside of hospital setting
	3.13: Number of antenatal care clients with tetanus vaccination	N/A	100%	33%	89%	N/A
	3.14: Number of antenatal care clients receiving preventive therapy for malaria	N/A	88%	44%	89%	N/A
	3.15: Number of antenatal care clients receiving syphilis screening	N/A	50%	22%	33%	N/A
	3.16: Number of antenatal care clients receiving urinary tract infection screening or treatment	N/A	88%	33%	67%	N/A
	3.17: Number of clients with identified maternal morbidities during post-natal care	N/A	75%	33%	78%	N/A
Newborn Health	4.1: Number of neonatal deaths	*# of neonatal deaths (0–28) at the facility level* Should be coupled with an indicator tracking neonatal death within the community	75%	44%	67%	Increased transparency in auditing practices surrounding neonatal death audits Training and capacity building for community health workers
	4.2: Number of stillbirths	N/A	75%	44%	67%	N/A
	4.3: Number of babies born low birth weight	Should be coupled with an indicator tracking malnutrition among pregnant women	75%	44%	78%	N/A
	4.4: Number of small and sick newborns receiving care	N/A	50%	22%	78%	N/A
	4.5: Number of newborns receiving post-natal care	*# of newborns receiving post-natal care within 7–10 days*	63%	22%	67%	N/A
	4.8: Number of neonatal deaths, disaggregated	N/A	63%	11%	67%	When reviewing data and making recommendations for health programs, consider the disaggregated indicator Leverage systems that capture newborn death and review these cases for cause of death Training and capacity building for of primary care providers
	4.9: Percentage of perinatal death reviews	French wording should be changed to “revue” as this is how it is reported in DHIS2	25%	22%	67%	In urban settings, national systems would need to be established to capture newborn death and review these cases for cause of death Training and capacity building of primary care providers Will need to be added to the DHIS2
	4.10: Number of newborns receiving Hepatitis B	N/A	0%	0%	22%	Will need to be added to the DHIS2 Extensive training and capacity building will need to be implementing for scale-up at the national level
	4.11: Number of newborns initiating breastfeeding early		75%	33%	56%	N/A
	4.12: Number of infants weighed at birth	*# of newborns weighed at delivery*	75%	11%	56%	N/A
	4.13: Number of babies registered	Distinguish between registration at the facility level and at the state level	63%	22%	67%	Civil registries to be integrated into hospitals and clinics Significant training and resources will need be implemented
	4.14: Number of newborns receiving treatment for possible severe bacterial infection (PSBI)	N/A	63%	0%	56%	N/A
	4.15: Number of newborns admitted	N/A	25%	11%	44%	N/A
	4.16: Number of newborns with morbidities identified during post-natal care	N/A	38%	0%	44%	Resources for primary care providers on the definitions for morbidity type Will need to be added to the DHIS2 Extensive training and capacity building of primary care providers
Child Health	5.1: Number of deaths of children under 5	N/A	88%	0%	44%	Training and outreach programs will need to be implemented at the health facility and community level Child health indicators to be integrated into routine service delivery as a specific area of its own, including for data collection
	5.4: Coverage of diarrhea treatment	N/A	50%	14%	44%	Child health indicators to be integrated into routine service delivery as a specific area of its own, including for data collection
	5.6: Percentage of children under 5 who are registered	The denominator of this indicator is not feasible since population level metrics are often unreliable in the DRC	25%	0%	56%	Child health indicators to be integrated into routine service delivery as a specific area of its own, including for data collection Significant resources and training will need to occur for this to be captured
	5.7: Number of children presenting with fever tested for malaria in endemic settings	*# of children under 5 who have been administered outpatient malaria tests, by age*	50%	14%	56%	Child health indicators to be integrated into routine service delivery as a specific area of its own, including for data collection
	5.8: Number of confirmed cases of malaria in endemic settings	N/A	50%	14%	56%	Child health indicators to be integrated into routine service delivery as a specific area of its own, including for data collection
	5.9: Percentage of confirmed malaria cases treated	N/A	50%	14%	56%	Child health indicators to be integrated into routine service delivery as a specific area of its own, including for data collection
	5.10: Coverage of DPT3	N/A	50%	14%	56%	Child health indicators to be integrated into routine service delivery as a specific area of its own, including for data collection
Adolescent health	6.4: Percentage of adolescents living with HIV who are currently receiving antiretroviral therapy, disaggregated	*# of adolescents living with HIV who are currently receiving antiretroviral therapy, disaggregated by age (less than 15 years; between 15–19 and equal or greater than 19 years)* Removal of the denominator	38%	38%	22%	Significant training and capacity development for frontline staff on adolescent health indicator reporting Will need to be added to the DHIS2
	6.5: Immunization coverage rate	*# of adolescents receiving the nationally mandated immunization, disaggregated by age (less than 15 years; between 15–19 and equal or greater than 19 years)* Removal of the denominator	50%	38%	44%	Significant training and capacity development for frontline staff on adolescent health indicator reporting Will need to be added to the DHIS2
SGBV	7.1: Number of rape survivors	N/A	88%	50%	78%	An anonymous code should be assigned to each survivor to avoid duplication of data
	7.3: Percentage of rape survivors receiving HIV post-exposure prophylaxis	Should be coupled with indicators capturing referrals and availability of supplies	63%	29%	67%	Significant training for the entire health care team Training on the new IAFM Inclusion of community leaders Inclusion of judicial system Training and outreach for community leaders Changes in the DHIS2 will need to be made to avoid duplicity in the data reporting Training in clinical management of HIV Increased availability/supply of PEP kits Coordination with the Justice System
	7.4: Percentage of rape survivors receiving emergency contraception	Should be coupled with indicators capturing referrals and availability of supplies	88%	38%	67%	Significant training for the entire health care team Training on the new IAFM Training and outreach for community leaders Inclusion of judicial system Changes in the DHIS2 will need to be made to avoid duplicity in the data reporting Coordination with the Justice System
HIV	8.1: Antiretroviral therapy coverage among people living with HIV, disaggregated	N/A	38%	38%	56%	Training in clinical management of HIV
	8.2: Percentage of exposed individuals receiving post-exposure prophylaxis	N/A	38%	38%	56%	Training in clinical management of HIV
	8.3: Percentage of donated blood units screened for HIV in quality assured manner	N/A	38%	13%	56%	Training in clinical management of HIV Might not be useful outside of hospital setting
Prevention of mother-to-child transmission	9.1: Percentage of antenatal care clients receiving syphilis screening and treatment	N/A	50%	25%	44%	Scale-up of these programs at the national level Training and capacity building of staff for service delivery and data capturing
	9.2: Percentage of antenatal care clients offered testing for HIV	*# of first-time antennal care clients who received pre-testing counselling for HIV*	50%	25%	56%	Training in clinical management of HIV
	9.3: Percentage of HIV-positive pregnant people receiving antiretroviral therapy	N/A	63%	25%	44%	Training in clinical management of HIV
	9.4: Percentage of all deliveries to HIV-positive mothers receiving antiretrovirals	*#of HIV-positive mothers who receive ART according to national protocol* Should be coupled with an indicator for the infant also receiving ART	50%	25%	44%	Training in clinical management of HIV
STI & RTI	10.1: Percentage of STI/RTI cases managed	*# of patients with STI/RTI accessing services who are diagnosed symptomatically, and counselled according to protocol* Distinguish between the number of cases and the number of cases “managed” STI and RTI cases need to be formulated as separate indicators	75%	15%	78%	Training on how to identify and report on STI/RTIs Training on the clinical management of STI/RTI cases

Italicized text refers to current description of indicator

^a^As mentioned in the body of the report, our stakeholders noted that data and trends of the different provinces across DRC is available and accessible on DHIS2 by all provincial members. Given that this information is accessible, coding the abortion indicators on DHIS2’s interface is crucial to protect, patients, health care providers, agencies and the provinces

^b^Service mapping: Mapping of service providers who provide safe abortion care—in their respective governorates—to the fullest extent of the law to enable the feasibility of collecting the abortion indicators

^c^As mentioned within the body of the report, the data and trends of the different health zones and provinces that are submitted on DHIS2 are universally accessible to anyone who has access to the HIS in the DRC

^d^IAFM guidelines: The Inter-Agency Field Manual on Reproductive Health in Humanitarian settings is a set of guidelines issued by normative bodies, particularly those of the World Health Organization, and incorporates specific evidence from, or examples about, the application and adaptation of global SRH or human rights standards in humanitarian settings

### (3) Existing systems and resources for collecting SRMNCAH indicators

The findings from our study indicate that systems used by international organizations are distinct from those of the health information systems (HIS) used by local organizations. Our results suggest that there is a harmonized nationally-endorsed reporting system for collecting SRMNCAH indicators in the DRC for services that are implemented at the national level. We provide a list of the available data collection systems by level of the health system structure and its use, reported by our stakeholders and frontline workers in Table 3.

**Fig. 2:**
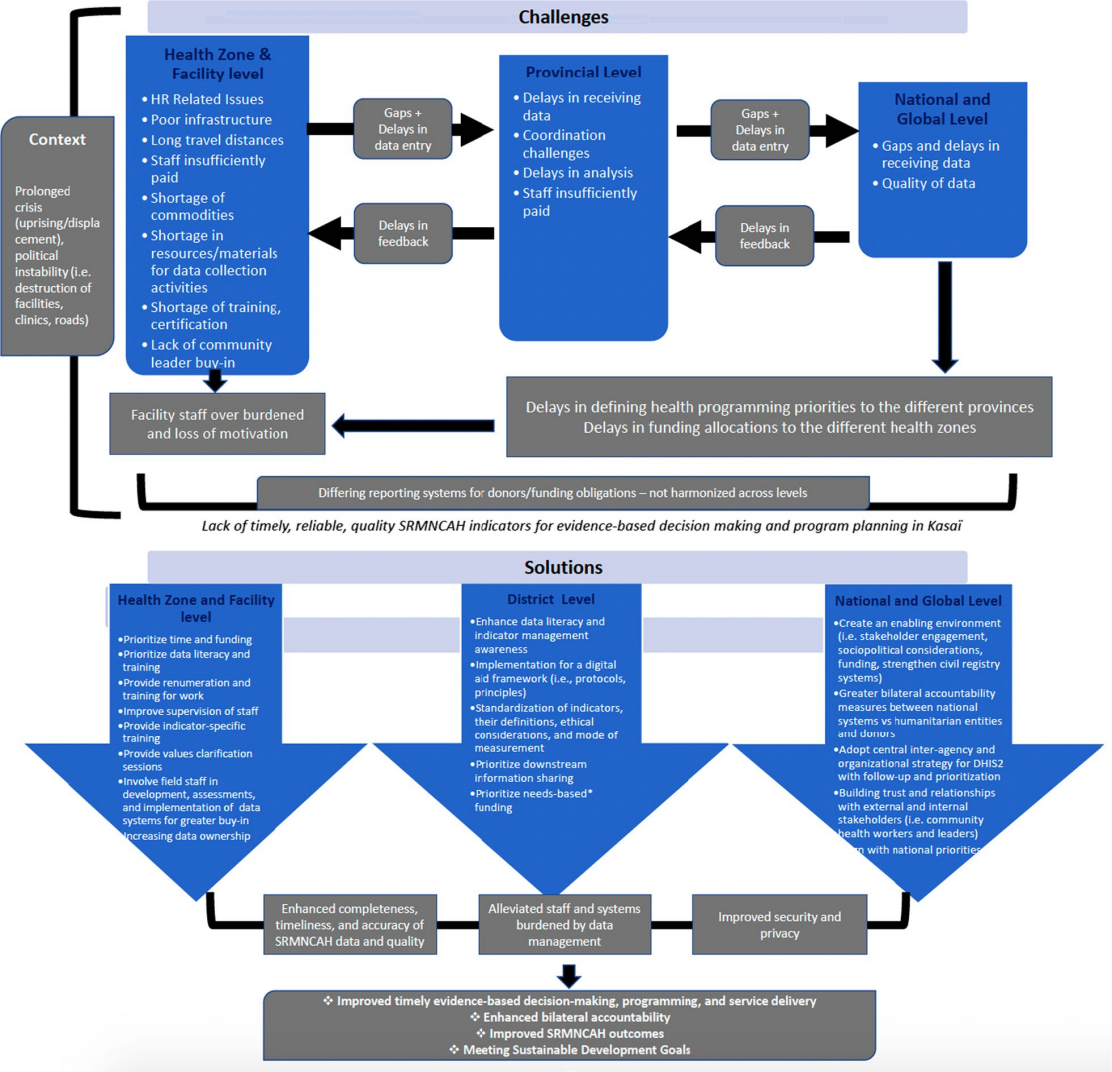
Challenges and solutions for timely, reliable, quality SRMNCAH indicator reporting at the facility, programmatic, and national and global levels in the DRC, reported by the study participants. *Backed up by the literature, our findings confirm that the matrix used for funding allocation to the health zones is incompatible with the needs

The health zone, provincial level, national level, and organizations in the Kasaï provinces report on a harmonized HIS, DHIS2, to capture information for certain SRMNCAH components. The findings also reveal that the structure of the health system and the well-defined roles of the national, provincial, health zone and facility levels, has enabled for a structured streamline of information and feedback loops. However, distribution of resources (i.e., internet, computers, infrastructure, data collection tools such as papers and pens) at the different levels of the hierarchy varied significantly. Due to scarcity of resources and weak infrastructure at the facility and health zone levels, upstream quality data distribution becomes more challenging. Finally, there are significant gaps in resources and systems that impede the input and analysis of timely and reliable SRMNCAH data (see Fig. 2).

### (4) Ethical considerations

The findings from this assessment suggest that across the health care system in DRC and among implementing agencies, data protection and confidentiality surrounding SRMNCAH data and research is prioritized; however, due to limited resources, data protection cannot always be assured. Health care facilities have basic protocols in place for data logging and protection, especially surrounding SGBV and HIV/AIDS data. Many of the agencies had adopted the requirements of the DHIS2; however, stakeholders at the provincial level vocalized concerns regarding data duplication, specifically concerning the indicators surrounding SGBV. Participants from all levels noted that reported cases of sexual violence are anonymously collected and coded at both the health center level and at the Ministry of Justice (if the case is brought there), as these two systems work in silo when addressing cases of sexual violence, leading to data duplication. As explained by a KI in Kinshasa, “We need to create a harmonized tool so we can all speak the same language. For example, in the field of violence, it’s multisectoral, the people from the protection sector are collecting, the health care people are collecting, the justice people are collecting. So, all of these domains, they are [collecting] about the same person. When someone is raped, they need psychological support, this is all centered around the same person. So, it takes [a harmonized system] …. the same person then makes a complaint, the justice department who is helping them, who managed their case, it’s all about the same person. So, a tool is really needed. A tool and active data collection.”

The findings from our interviews with both KIs and FGDs suggest that although there is both demand and supply for abortions (through illegal channels), primary care providers mask/underreport indicators related to abortion care in fear of retribution. As a stakeholder explained, “Complete abortion care… you can have it partially. Since, first of all, abortion is illegal. As it’s illegal, I would say that this information is concealed, since if we trace this information back to the source, we could identify the person who logged it as being complicit [in the abortion]. I believe that this is information that is still being masked, I would say.” Thus, if abortion-related indicators are incorporated, a degree of anonymity must be given to staff at the health facilities and coded through the DHIS2 to protect primary care providers and women from potential retribution. Our findings also indicate that values clarification and attitudes transformation (VCAT) sessions and training on the IAFM are warranted.

## Discussion

The findings from our multi-methods assessment suggest that the DRC has a robust and dynamic HIS system that can be leveraged and improved to ensure the feasibility of collecting a core set of indicators for monitoring and evaluating SRMNCAH services and outcomes in the humanitarian context in the Kasaï provinces and across DRC more broadly. The NHIS data quality score increased from 25% in 2018 to 40% in 2021 [16]. However, results from our assessment, supported by the literature, indicate that despite having a centralized HIS system and recent improvement of data quality, there lacks a harmonized list of indicators for agencies to report against [10]. Much of this burden falls onto low resourced health facilities at the local level [17, 23]. In order to mitigate this, overwhelmingly, the stakeholders felt it was important to develop an evidence-based, context-specific, core set of SRMNCAH indicators to be collected in DRC in conjunction with strengthening and investing in health facility data management systems at the health zone or district level to ensure widespread coverage and needed granularity of information [24].

A harmonized list of a core set of SRMNCAH indicators would alleviate the reported manual and human resource burden from the health facility, health zone and community levels [25, 26], and also improve data analysis and reporting at the provincial and operational level [17]. However, this shift to a core set of indicators requires a multi-pronged effort and buy-in across trans-disciplinary agencies working towards the same goal with horizontal and transparent communication channels. The outlined recommendations are steppingstones for SRMNCAH services and outcomes monitoring and evaluation in humanitarian settings. The recommendations also highlight work that can be done by data managers, communities, donors, and humanitarian actors in creating an enabling environment for quality SRMNCAH data and evidence-based decision making. In Fig. 2. we outline factors that are supported by the literature that need to be implemented and addressed at the global, national, programmatic and facility levels to increase the feasibility of current indicator reporting practices and the quality of SRMNCAH data reporting [27–32].

The findings from this assessment indicate that continued armed conflict and looting, limited budgets and ownership, infrastructure challenges, and varying, incompatible policies prohibit the development of national priorities and guidelines for improving the reporting of SRMNCAH indicators. These issues are exacerbated in refugee and internally displaced populations more specifically, which has proven to be challenging in the context of the Kasaï provinces. Reports suggest that donors reacted by creating and implementing projects in search of rapid results and data to document them [33]. Both the literature and the findings of this assessment show that this reactive approach has led to multiple donor coordination mechanisms and project management units and, critically, a duplication and waste of resources for supervision, training, and technical assistance [33]. This created a significant burden on the time of staff at district and health facility levels to attend training seminars, respond to supervisory visits, and meet reporting requirements [33]. Further, many humanitarian agencies are financially supporting health zones under a performance-based contracting scheme [34], with clear emphasis on health facility reporting [25].

To improve SRMNACH services and outcomes in humanitarian settings, WHO, in collaboration with partner agencies, supported the development of a ‘Data and Accountability Roadmap for Improving Data, Monitoring and Accountability for SRH in Crises’ [30]. This Roadmap responded to the need for collecting, aggregating, and making accessible clear and consistent data. The response aligns with the SDGs, the ‘Every Women Every Child Strategy’, and the commitments made at the 2017 Family Planning Summit to address data gaps. The Roadmap combined with data literacy and indicator management awareness, digital aid frameworks and amendments of laws and regulations should be prioritized at the national and programmatic level to create an enabling environment to reduce duplicity, and improve communication channels and overarching SRMNCAH data quality [35].

The results from our assessment also indicated that the infrastructure in the Kasaï provinces and access to resources is weak and varied by health zone. This finding is consistent with the lack of human, financial, and material resources observed at the country level, where 4 out of 10 health facilities do not have staff designated for data verification, and staff of more than 90% of health facilities have not been trained in recording, compiling and/or reviewing data quality [16]. A functioning HIS requires skilled and motivated personnel, access to basic information and communication technologies, electricity, and roads [18]. All these factors are likely to be negatively impacted by armed conflict as seen in the DRC through direct attacks and looting, as well as indirectly due to insecurity, reduced mobility, and limited availability of financial and human resources [36]. Strategies to improve routine facility data collection and reporting that are tailored to conflict affected settings can contribute to improve the effectiveness of the system [17], including investing in innovative solutions to overcome the barriers associated with weak infrastructure and limited resources [37]. It would also be important to ensure that those at the facility-level have the needed training and skills necessary to provide services associated with the minimum initial service package (MISP) and have the training to reliably capture and analyze data. The findings also indicate that there needs to be some level of reflection on the principle of data minimization^7^ to protect populations in humanitarian settings [38].

Stakeholders were clear that collecting the SRMNCAH indicators that required a population-level denominator may not be often feasible, which could particularly be exacerbated in conflict affected areas in the DRC. A recent study found that data completeness and accuracy of reproductive, maternal, newborn and child health (RMNCH) data and difficulties of calculating population denominators were among the most common concerns among RMNCH key stakeholders in the DRC [17]. Furthermore, the country’s most recent census was organised in 1984 and its civil registration system is too weak to provide reliable population-level denominators [19]. Investing in characterised health facility data can potentially provide crucial disaggregated and timely information for program planning purposes and for evidence-based resource allocation in conflict settings that complement nationwide population-based surveys conducted on average every 5 years [26, 39].

The findings from our study indicates that facilities need to recruit staff with a data collection and processing background (demographers, statisticians, data scientists) as well as provide regular significant pre-service and in-service training of HIS staff in the DRC (from health centers to the central level). It is important to identify ways to support organizations that are currently providing SRMNCAH services with their data collection methods through additional staffing or training of existing staff. Given that all capacity building occurs through the MoH, training efforts should also occur through the MoH. Our findings indicate that given the prevalence of staff recycling, increasing staffing and training specifically for data collection and supervision at the health facility level is paramount to alleviate burden and enhance motivation among staff members to improve quality of data. Finally, the proposed indicators should be introduced with a toolkit that facilitates data collection by explaining the link between reporting and program improvement to enhance buy-in across staff members tasked with data collection. The trainings, policy changes, and resources described above would work to ensure accurate and quality data collection across the DRC.

There is also the need for the standardization of definitions and strata and a need for the digitalisation of data collection from health centres in order to develop a multi-sectoral integrated data system. For instance, age group categories in the current HIS vary sometime by indicator (i.e. < 5 years, 5 years and above; < 14, 15 years and above, etc.). Such stratification might not allow for the analysis of certain adolescent SRH-related indicators or factors associated with maternal health, such as the use of mosquito nets among adolescent pregnant women and intermittent preventive treatment of malaria in pregnancy. Likewise, current paper-based data collection at the health centre level might introduce data entry errors at the health zone level.

## Strengths and limitations

The strategies recommended by Guba [40] were used for evaluating the trustworthiness of the data in this assessment. The four components of trustworthiness outlined by Guba include credibility, dependability, transferability, and confirmability [40]. Prolonged engagement, triangulation, member checks, peer-debriefing with the study team in both DRC and Canada were used to ensure the credibility of the data [41]. The multi-methods design of this study enabled the integration of data from multiple sources, allowing the study team to validate and outline discrepancies between the data. Qualitative data was used to explore and explain the quantitative findings, with the findings from the facility assessments also validating (or in some cases dispelling) key themes identified in analyses of FGDs and KIIs. The facility assessments provided important findings relating to current management SRMNCAH monitoring and evaluation systems as well as the availability and distribution of specific resources. Despite these strengths, however, our study was limited by challenges with language and translations, documentation, and infrastructural challenges. For example, as all interviews were conducted in the local French language and translated by researchers in Canada, saying or words might have been mistranslated/misinterpreted due to regional language differences. The positionalities of the research team members undoubtedly influenced the participant-researcher interaction as well as our interpretation of data collected. Through memoing and regular debriefings, we attempted to reflect on and understand these dynamics, thereby enhancing the credibility and trustworthiness of the findings.

## Conclusion

The findings from this multi-methods feasibility assessment suggest that vast differences in feasibility exist in collecting the proposed list of core SRMNCAH indicators across the different Kasaï provinces and different levels of the health system. Notable priority gaps exist in service domains, particularly within the domains of adolescent health and abortions, since under 18 populations and unsafe abortions incur high risks. The gaps in these areas render the collection of certain indicators unfeasible, and have been recommended for removal in order to safeguard populations from potential harms associated with that domain. Representatives from a variety of institutions noted that leveraging and further investing in current national systems and resources, such as the DHIS2, will ensure the feasibility of collecting this core set of indicators for monitoring and SRMNCAH services and outcomes in humanitarian settings in the DRC. The findings of this study will also feed into humanitarian programs nationally and in other provinces in the DRC to aid in understanding the nuances in feasibility of indicator collection, with the eventual goal of feeding into a broader set of core SRMNCAH indicators to use in humanitarian settings. The majority of the participants were in consensus that multi-pronged, transdisciplinary interventions and investments at the national and international levels would be required to increase the feasibility of collecting quality and timely SRMNCAH data. Indeed, stakeholders in the DRC were clear that this core set of SRMNCAH indicators can only be useful if buy-in from the global community to harmonize and coordinate data collection efforts and relevant indicators’ reporting requirements is ensured.

## Data Availability

Available upon request.

## Abbreviations

BEmOC: Basic emergency obstetrical care
CEmOC: Comprehensive emergency obstetrical care
DHIS2: District Health Information System 2
DRC: Democratic Republic of Congo
FGD: Focus Group Discussions
HIS: Health Information System
HIV/AIDS: Human immunodeficiency virus/acquired immunodeficiency syndrome
IAFM: Inter-Agency Field Manual on Reproductive Health
IDP: Internally displaced persons
KI: Key informant
KII: Key informant interview
KMC: Kangaroo mother care
MISP: Minimum Initial Service Package
MoH: Ministry of Health
NGO: Non-governmental organization
NHIS: National Health Information System
PAC/CAC: Post abortion care/comprehensive abortion care
PEP: Post-exposure prophylaxis
RMNCH: Reproductive, maternal, neonatal, and child health
RP2: Research Project Review Panel
SRH: Sexual and reproductive health
SRMNCAH: Sexual, reproductive, maternal, neonatal, child, and adolescent health
U5MR: Under-5 mortality rate
UN: United Nations
UNHCR: United Nations High Commissioner for Refugees
UNICEF: United Nations Children’s Fund
VCAT: Values clarification and attitudes transformation
WHO: World Health Organization

## Footnotes

1 Sphere Association. The Sphere Handbook Humanitarian Charter and Minimum Standards in Humanitarian Response. 2018. Available from: www.practicalactionpublishing.org/sphere.

2 Inter-Agency Standing Committee. Guidelines for integrating gender-based violence interventions in humanitarian action: reducing risk, promoting resiliewnce and aiding recovery. 2015. https://interagencystandingcommittee.org/working-group/iasc-guidelines-integrating-gender-based-violence-interventions-humanitarian-action-2015.

3 See Ref. [10].

4 A more detailed description of this projects’ methodology can be found in the report for the WHO.

5 LIVES Model developed by the World Health Organization: includes a first-line support that involves five simple tasks; (1) **Listen** to the woman closely, with empathy, and without judging; (2) **Inquire about needs and concerns**, assess and respond to her various needs and concerns—emotional, physical, social and practical (eg., childcare); (3) **Validate**, show her that you understand and believe her. Assure her that she is not to blame; (4) **Enhance Safety**, discuss a plan to protect herself from further harm if violence occurs again; and (5) **Support** her by helping her connect to information, services and social support.

6 The **Maputo Protocol**, also known as the Protocol to the African Charter on Human and Peoples’ Rights on Women in Africa, is an international human rights instrument established by the African Union that went into effect in 2005. It guarantees comprehensive rights to women including the right to take part in the political process, to social and political equality with men, improved autonomy in their reproductive health decisions, and an end to female genital cutting/mutilation.

7 Data minimization principle: the data collected is adequate, relevant and limited to what is necessary in relation to the purposes for which they are processed.
